# Medical News and Items

**Published:** 1878-01

**Authors:** 


### Medical News and Items.

Origin of the Term Syphilis.—Upon this subject the *Louisville Medical News* of December 1, 1877, publishes the following communication from Dr. James Nevins Hyde, of Chicago:

"In your issue of the 3d instant, I find a paragraph, copied from the *London Medical Press and Circular*, in which it appears that Dr. L. P. Yandell, Jr., has offered a new theory as to the origin of the term syphilis. According to the latter, the first popular name of the disease was *morbus civilis*, i. e., the "citizens' or town disorder"—terms used "by the lower classes, and especially among the rustics." It is suggested the word *morbus* was dropped for the sake of brevity, and that the subsequent transformation of *civilis* into syphilis is not remarkable, in view of the fact that chancre was once spelled "shanker," and scrofula "scrophula."

"This quite ingenious and plausible explanation suggests the inquiry whether it can be sustained by the facts already known concerning the history of the word. We are led to ask, *what* rustics first employed the term in its original form? Certainly not those of the old Roman Campagna, for Hippocrates, Celsus and their immediate successors never thus designated the disease, although they had frequent need of a periphrasis for that purpose, and would surely have been careful to append the popular name had such existed. Even if they had failed to do this, such a popular term would have almost inevitably crept into the writings of such authors as Plutarch, Martial and Horace, all of whom refer to the disease in unmistakable phraseology.

"The truth is that the word syphilis never made its appearance in literature of any kind before Hieronymus Fracastorius, the Veronese physician, in 1521, wrote his famous poem in which the herdsman of King Alkithous, named Syphilus, is smitten with the disease by Apollo, as a penalty for his offense against the god. According to G. Fallopia, the name was derived from the Greek words which are commonly supposed to explain the ignoble origin of the malady. Twenty-five years later, Fracastor employed the term in his second work upon the same subject.

"Now, since it is admitted that Fracastor first employed the word syphilis, it is fair to ask, if the word was not actually coined by him, whence did he borrow it? He was at the time one of the chief luminaries of the Italian school. Did he first hear the mis-pronunciation upon the lips of the Italian peasants around Verona?

"It may be safely concluded that the most illiterate of Italian rustics would never pronounce in such a manner as to change the sound represented by v into that of ph. He might, indeed, under some circumstances, convert ph into f, but his unconscious law of metastasis would never permit the reverse of this. The Italian of to-day spells syphilis *sifilis*, and transforms the initial ph of all words of Greek origin into f; but his *civile* of to-day is accepted from his Roman ancestors without change, and he has never suffered the loss of the v of his forefathers. Even the Frenchman, who has adopted the Greek ph in its original form, writes *sulfure* and *calife* for sulphur and caliph; while he and his southern neighbor have alike softened the b of *habere* and similar words, into the v of *avoir* and *avere*.

"There is a law to be observed even in the aberrations of the lips and tongue, which the English sailors demonstrated when they affectionately christened their 'Bellerophon,' the 'Bully Ruffian.'

"In short, while the natives of Southern Europe have again and again transformed b into v, and ph into f, it is altogether improbable that they have ever converted ph into v, and physically impossible for them to exchange v for ph, so that *civilis* in their lips could be represented by *syphilis*.

The letters y p h in syphilis point as unmistakably to its Greek origin, as its mucous patches to a primary lesion. Fracastor was, it is true, under the influence of the astrological ideas of the physicians of his day; but he was an erudite author, and drew an eminently graphic picture of the disease as he saw it. He was quite capable of coining the word which he introduced into medical literature, and which, we trust, may survive the disease itself, but whether he coined or borrowed the term, its parentage in the Greek language is as distinct as the source of hereditary syphilis.

"And I am inclined to believe that my good friend, Dr. Yandell, will, after consideration, accept my conclusions."

List of Persons who Passed the Examination of The Illinois State Board of Health, held at Chicago, November 1, 1877, fourteen candidates: James P. Pearson, Fairbury, Ills.; Herbert Wheeler, Grant Park, Ills.; Max. Muffas, Wheeling, Cook Co., Ills.; Elias Miller, Fairbury, Livingston Co., Ills.; Royal McShea, Owaneco, Christian Co., Ills.; C. C. Watson, Chicago, Ills.; Edward Armstrong, Northville, LaSalle Co., Ills.

The following were successful at a similar examination held at Cairo, November 15, 1877, forty-two applicants: D. L. Gebhart, Metropolis, Massac Co., Ills.; Julius C. Gebhart, Massac Creek, Massac Co., Ills.; A. R. Ransom, Grantfork, Madison Co., Ills.; R. P. Lightfoot, Carbondale, Ills.; D. C. Asher, New Burnside, Johnson Co., Ills.; John W. Jeffries, Sauer, Jefferson Co., Ills.; Thos. J. Green, Phillipstown, Ills.; E. H. Wheeler, Gillsburgh, Jackson Co., Ills.; John Keese, Dongola, Union Co., Ills.; John W. Ross, Fitts Hill, Franklin Co., Ills.; H. L. Burnett, Raleigh, Ills.; W. S. Harvey, Beverley, Adams Co., Ills.; Henry C. Buck, Olmsted, Pulaski Co., Ills.; Wm. P. Sutherland, Golconda, Ills.; Geo. W. Hendershott, Mill Shoals, White Co., Ills.; John Wm. Mott, Villa Ridge, Ills.; Ch. H. Cristoffe, French Village, St. Clair Co., Ills.

The following passed successfully the examination held at Galesburg, December 6, 1877,—eighteen candidates: Wm. S. Williamson, Rio, Ills; J., Parker, Ipava, Ills.; Z. T. Harvey, Brooklyn, Schuyler Co., Ills.; Boggs, M. C., Gerlaw, Ills.; F. F. Noyes, Ray, Schuyler Co., Ills.; Wm. Parker, Huntsville,

Schuyler Co., Ills.; Chas. M. Verbrees, Murrayville, Morgan Co., Ills.; Thos. A. Scott, Kirkwood, Warren Co., Ills.

At this meeting a resolution was unanimously adopted by the Board to the effect that the diplomas of the St. Louis Homeopathic College, of the Physio-Medical College and the Physio-Eclectic Medical College, of Cincinnati, Ohio, would not be recognized.

The following resolutions were also passed unanimously:

*Resolved*, That on and after July 1, 1878, the Board will not consider any medical school in good standing that holds two graduating courses in one year.

*Resolved*, That on and after July 1, 1878, the Board will not recognize the diplomas of any medical school which does not require of its candidates for graduation the actual attendance upon at least two full courses of lectures, with an interval of six months or more.

At the annual meeting of stockholders of the Chicago Medical Press Association, held December 4, 1877, Drs. J. P. Ross, E. Ingals, and J. H. Etheridge, were elected to fill the vacancies created by the expiration of the term of office of Drs. Ross, Bridge and Etheridge. The Treasurer's report showed that the Association was able to sail along smoothly in spite of hard times and slow payments.

The Librarian reported that the collection contained to date 526 bound books, and about as many miscellaneous pamphlets; 75 reports of hospitals, asylums, etc.; 200 announcements and catalogues, and 6,400 copies of medical journals. The whole collection, with the exception of 70 books purchased, has been acquired by donation, and by exchange with the Journal and Examiner, and with the Library.

The system of cataloguing and indexing by cards has been introduced during the past year, and about 5,000 cards have been, so far, written and are now in use.

The system of circulation of journals introduced several months ago, has worked satisfactorily, and quite a number of members have availed themselves of its advantages.

THE PHYSICIAN'S HAND-BOOK FOR 1878, by Wm. and A. D. Elmer, needs no further recommendation than the statement of its having attained the age of twenty-one years. It is a *minor* no more, and can well take care of itself.

The editors inform us of their intention to entirely re-write the next edition. We hope they will not materially change the character of a book which has proved so useful to the profession; but we would suggest they might leave out the list of diseases, and that of the remedial agents. The omission of these lists will be no loss to any practitioner, but lighten the book materially.

DR. LEWIS A. SAYRE, under date of Oct. 15, 1877, addresses a communication to the editor of the *Phila. Med. Times*, in which he refutes the charge of plagiarism preferred against him in the *St. Louis Clinical Record*, as follows:

*Charge* 1st. "Dr. Sayre's hip-joint splint was invented by Dr. Davis." To refute this I refer you to the "Transactions of the American Medical Association" for 1860, pages 505 to 508, and by referring to the Patent Office at Washington, "Synopsis of Specifications," No. 35,303, you will see that Dr. Davis took out a patent for his splint, which you will observe in the specifications is entirely different from mine, which was given to the profession, as well as its various modifications and improvements, as soon as tested and proved to be useful. I also refer you to my "Orthopedic Surgery and Diseases of the Joints," Appleton & Co., 1876, pages 260, 261, to prove the falsehood of this first charge.

*Charge 2d.* "Dr. Sayre's plaster-of-Paris jacket was invented and first applied by Dr. Bryan, of Lexington, Ky."

*Answer.* See my report on Pott's Disease, "Transactions American Medical Association" for 1876, page 585, where you will see full justice has been done to Dr. Bryan; also *Richmond and Louisville Medical Journal* for May, 1877, page 418; also my recent work on "Spinal Curvatures and their Treatment by Suspension and the Plaster-of-Paris Bandage," Smith, Elder & Co., London, Eng., 1877, page 14. Any honest man reading these three references, I think, will never again repeat this charge.

*Charge 3d.* " Dr. Sayre's method of self-suspension in rotary lateral spinal curvature was invented by Dr. Benjamin Lee, of Philadelphia."

*Answer.* See my work on spinal curvature above referred to, Smith, Elder & Co., London, page 93. For fear that you may not be able to obtain the book in this market at present, I will quote the sentence on page 93 to which I refer:

"The late Prof. Mitchell, of Philadelphia, used to treat cases of lateral curvature by suspending them under the arms, and causing them to suspend themselves by the hands. But Dr. Benj. Lee, of Philadelphia, was the first person who caused his patients to practice *self-suspension*, by climbing up a rope which passed over a pulley and was attached to the patient's head by straps passing under the chin and occiput." I think this answers that charge.

*Charge 4th.* "Dr. Sayre's Lectures on Orthopedic Surgery were by Dr. Louis Bauer, formerly of Brooklyn, New York, now of St. Louis."

*Answer.* By referring to the preface of my book on "Orthopedic Surgery and Diseases of the Joints," Appleton & Co., New York, 1876, it will be seen that the book was published from stenographic notes of my lectures in Bellevue Hospital Medical College, session of 1874-75, taken at the time by Dr. Wesley M. Carpenter, of this city. Most of the lectures were upon cases presented at the time in the lecture-room, and which Dr. Bauer could never have seen, as he at the time lived in St. Louis. The statement is, therefore, too absurd to demand any further notice. The general charge of plagiarism in the last sentence quoted from the *Record*, not being *specific*, cannot be *specifically* refuted, but to it I make a general denial.

THE WARREN PRIZE.—The Warren Prize Committee, consisting of the visiting physicians and surgeons of the Massachusetts General Hospital, have awarded the prize of the present year, amounting to $371.41, to E. O. Shakspeare, M. D., of

Philadelphia, for an essay on "The Healing of Arteries after Ligation."

The committee also announce that the subject for 1880 will be, "Original Observations in Physiology, Surgery and Pathological Anatomy."

Essays should be forwarded to the resident physician, Massachusetts General Hospital, Boston, on or before February 1, 1880. The amount of the prize will be $400.

The conditions of the prize lately awarded were similar to those given in the announcement, the object, we understand, being to stimulate original researches. As evidence of the success of the plan to leave to the competitors the choice of a subject within certain limits, it may be mentioned that the number of essays presented was large. We learn that a dissertation on Pneumono-Dynamics and one on Certain Points on the Physiology of the Nervous System were highly praised by the committee for their merit. A third, on Bone, was much admired for the superb illustrations which accompanied it and the great labor which its prepararion evinced, particularly that portion devoted to Dentine.

The Illinois Charitable Eye and Ear Infirmary (cor. Peoria and W. Adams streets) has lately been enlarged by the erection of a three-story wing on the Peoria street front. This addition may be called the dispensary department of the infirmary, for its main part is fitted up for the reception and treatment of the out-door patients. The entrance from Peoria street leads by a small vestibule into a spacious reception room, where from one hundred to one hundred and fifty patients can easily be seated. The second floor is divided up in a room for the treatment of the ear patients, a room for the treatment of eye patients, and an ophthalmoscopic room. The treating rooms are large and high, supplied with warm and cold water; and many windows reaching from the floor to the ceiling, admit so much light from north and west, or north and east respectively, that the light is sufficient even on the cloudiest day. The dark room, in direct communication with the eye room, is provided with all the facilities for ophthalmoscopic examinations, and large enough to allow eight persons to make such examinations simultaneously.

The third floor is given up entirely for an operating and clinical lecture room, with which a smaller room is connected for the accommodation of patients waiting for the operation, or to rest and recover from the effects of the narcosis after the operation. A large sky-light above the operating table, furnishes a beautiful light to perform the most delicate operations. A broad and easy stairway, broken by several landings, leads from the first floor to the top of the building. The operating room communicates by a hall-way with the second floor of the main building, on which floor the rooms for operative cases are located.

By this arrangement patients can most easily and safely be removed from the operating table to their rooms. The second floor also has a communication with the main building, for the use of the surgeons and officers of the infirmary. But the dispensary patients are strictly prohibited to enter the main building, whose quiet and cleanliness is thus not disturbed by the daily rush of a noisy crowd of patients.

In its present form, the infirmary is probably the completest and finest building of its kind in this country, and Illinois may well be proud of this charity.

The State Board of Health and its Medical Examinations.—We have taken pains to carefully examine the method pursued by the members of the Illinois State Board of Health in examining non-graduates in medicine who apply for a license to practice; and have also critically read the entire list of all questions proposed to such candidates on the printed papers. We felt that the profession at large was concerned to such an extent as to warrant the scrutiny, and, as a result, we have no hesitation in declaring that the tests are ample, and that the standard is such as would do credit to all the medical schools of the State.

